# Supplementary material for: Acquired cancer resistance to combination immunotherapy from transcriptional loss of class I HLA
Source: Nat Commun. 2018 Sep 24;9:3868. doi: 10.1038/s41467-018-06300-3 (PMC6155241; doi:10.1038/s41467-018-06300-3)
Supplement: Supplementary file 1 — Supplementary Information [file 41467_2018_6300_MOESM1_ESM.pdf]

Supplementary Information

Paulson et al, Acquired Cancer Resistance to Combination Immunotherapy from Transcriptional Loss of Class I HLA

Supplementary Figures: 12

Supplementary Tables: 4

Supplementary Data: 4 (separately attached)

Supplementary Figures:

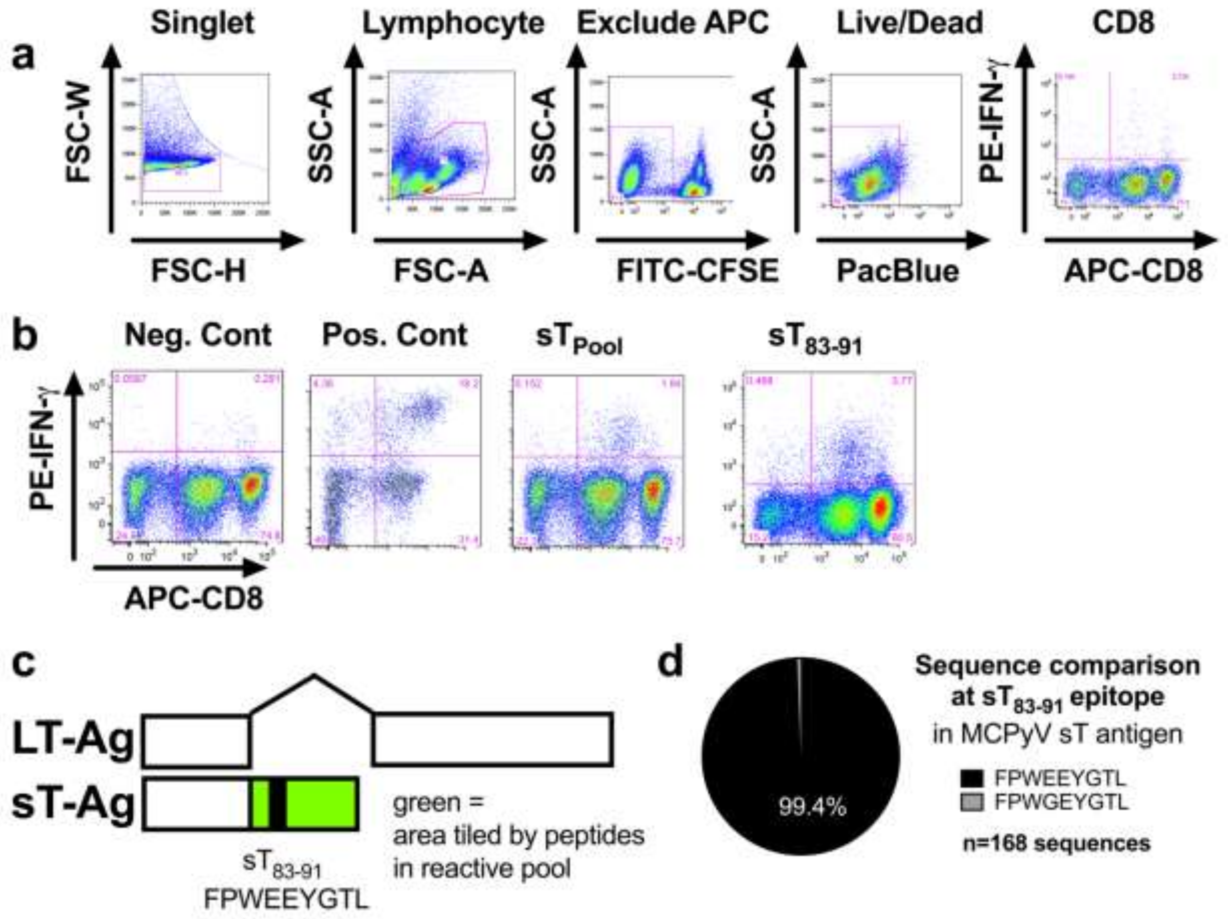

Supplementary Fig. 1:
A novel HLA-B*3502 restricted epitope in MCPyV small T antigen.
a and b Representative data from tumor infiltrating lymphocytes from a HLA-B*3502-expressing MCC patient. Tumor infiltrating lymphocytes were expanded for two weeks with cytokine support, and then co-cultured with CFSE labeled autologous PBMC used as antigen presenting cells plus candidate peptides. Reactivity to peptide was evaluated with interferon-gamma intracellular cytokine staining. **a** Gating Strategy: from left to right: singlets were gated in, then lymphocytes gated in, then CFSE-labelled APCs gated out, then live cells gated in, and $CD8^{+}$ expression used for final gating. **b** Response to candidate peptides: From left to right: IFNgamma-expressing (y axis) tumor infiltrating $CD8^{+}$ T cells (x axis) in response to no stimulation (negative control), PMA-ionomycin (positive control), peptide pool containing 27 13-mers overlapping by 9 tiling across unique C-terminal domain of MCPyV sT-Ag, and peptide corresponding to minimal epitope $sT_{83\text{-}91}$ **c** Schematic: location of epitope (MCPyV-$sT_{83\text{-}91}$) in the C-terminal "unique" domain of MCPyV small T antigen oncoprotein is shown. **d** MCPyV-$sT_{83\text{-}91}$ amino-acid sequence comparison: There were 168 available sequences in National Center For Biotechnology Information (NCBI). 167/168 (99.4%) of the available sequences conserved the FPWEEYGTL sequence across MCPyV isolates.

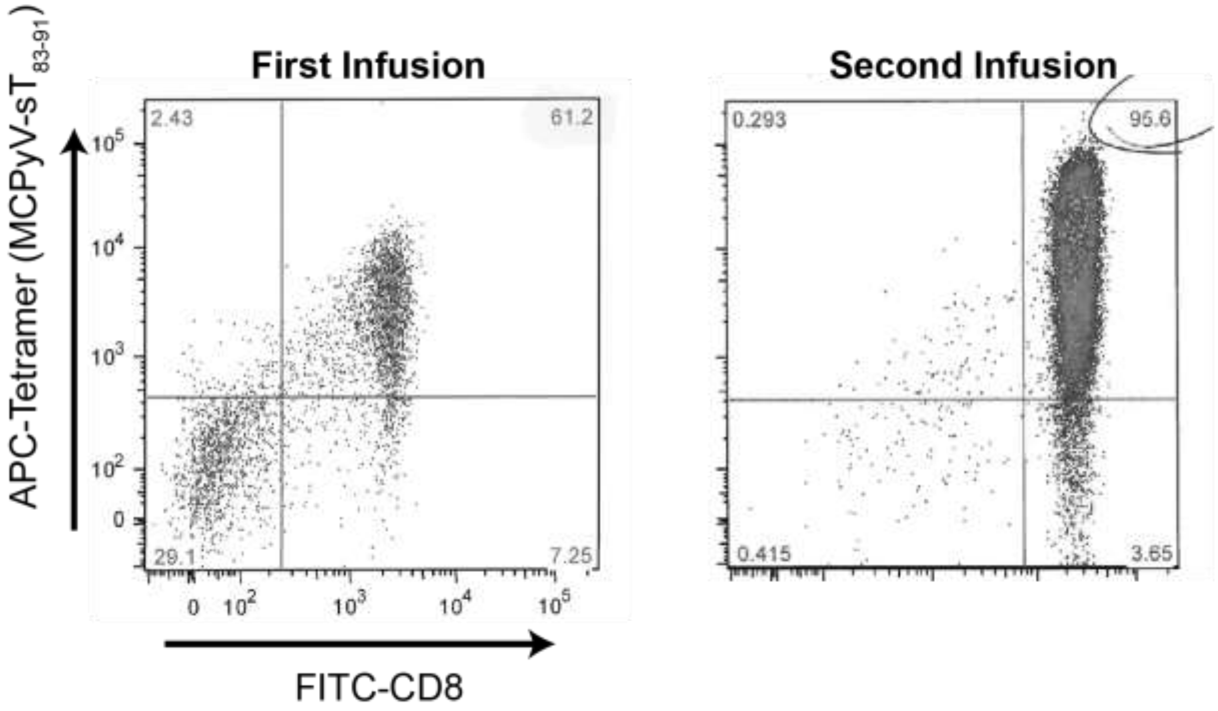

Supplementary Fig. 2:
Composition of infused MCPyV-specific CD8^{+} for T cell products
Binding of infused CD8 T cells (x-axis) to a tetramer of HLA B*3502 containing the MCPyV-sT$_{83-91}$ tetramer (y axis) in the 1st and 2nd infusions for patient 2586-4. The percentage of cells in each quadrant is shown**.**

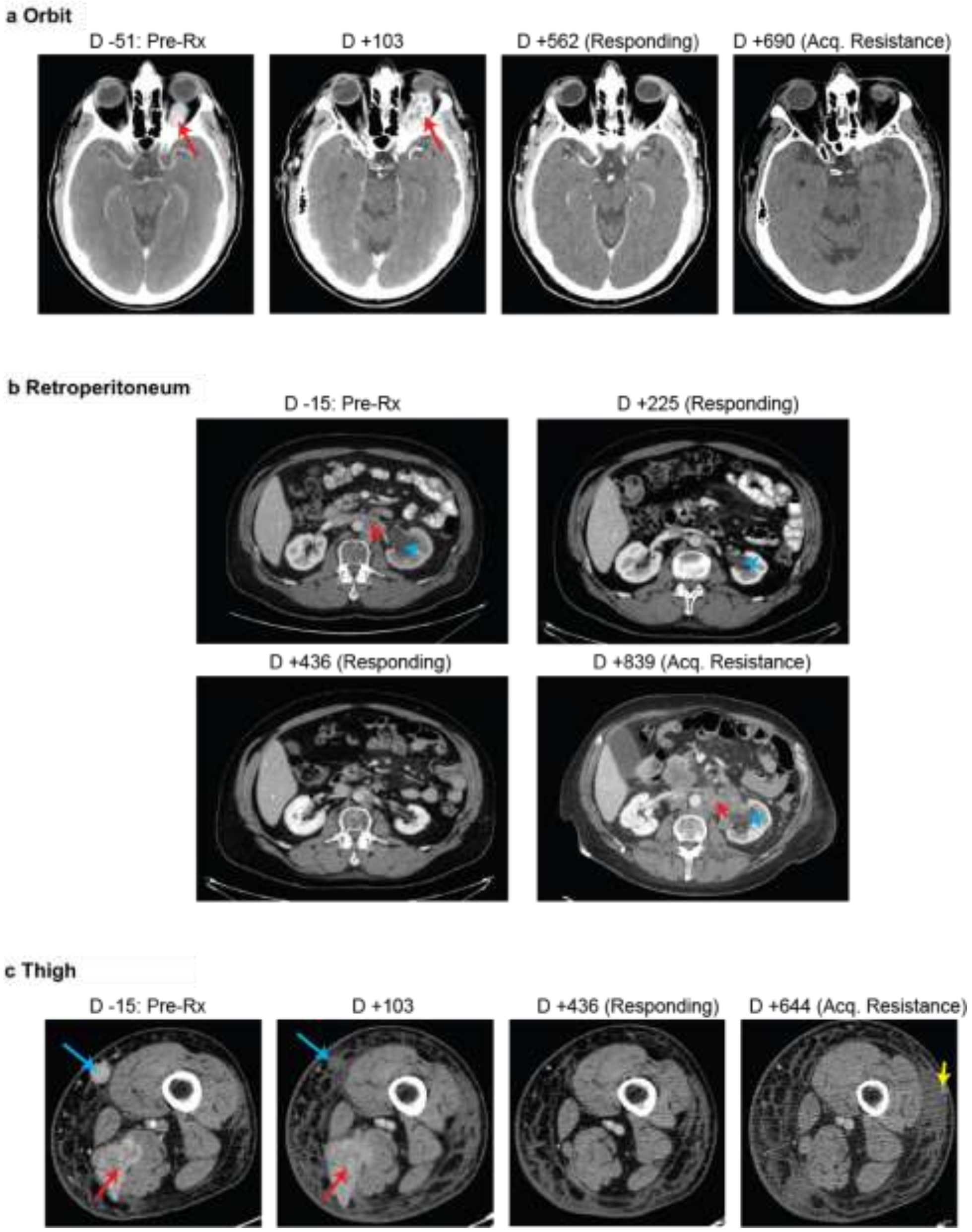

Supplementary Fig. 3:
Representative Clinical Images.
Images from discovery patient (2586-4). Representative slices from CT imaging from the patient's treatment course are shown. See Fig. 1 for a summary of the patient's tumor burden over time and treatment. Patient had multiple additional metastatic sites that are not pictured. **a** Orbit: A highly symptomatic radiation refractory left orital lesion initially expanded during the treatment period and then slowly regressed after D+103 (addition of ipilimumab), eventually fully regressing with no recurrence. **b** Retroperitoneum: Patient with bulky retroperitoneal disease (red arrow) that had been treated several previous times with radiation therapy and was causing hydronephrosis (blue arrow). Disease slowly regressed over the period of approximately one year, before recurring at the time of acquired resistance. **c** Thigh: 2 representative lesions are shown (red and blue arrows), both of which resolved during time of immunotherapy response. A new third lesion developing at time of recurrence/acquired resistance is also pictured (yellow arrow).

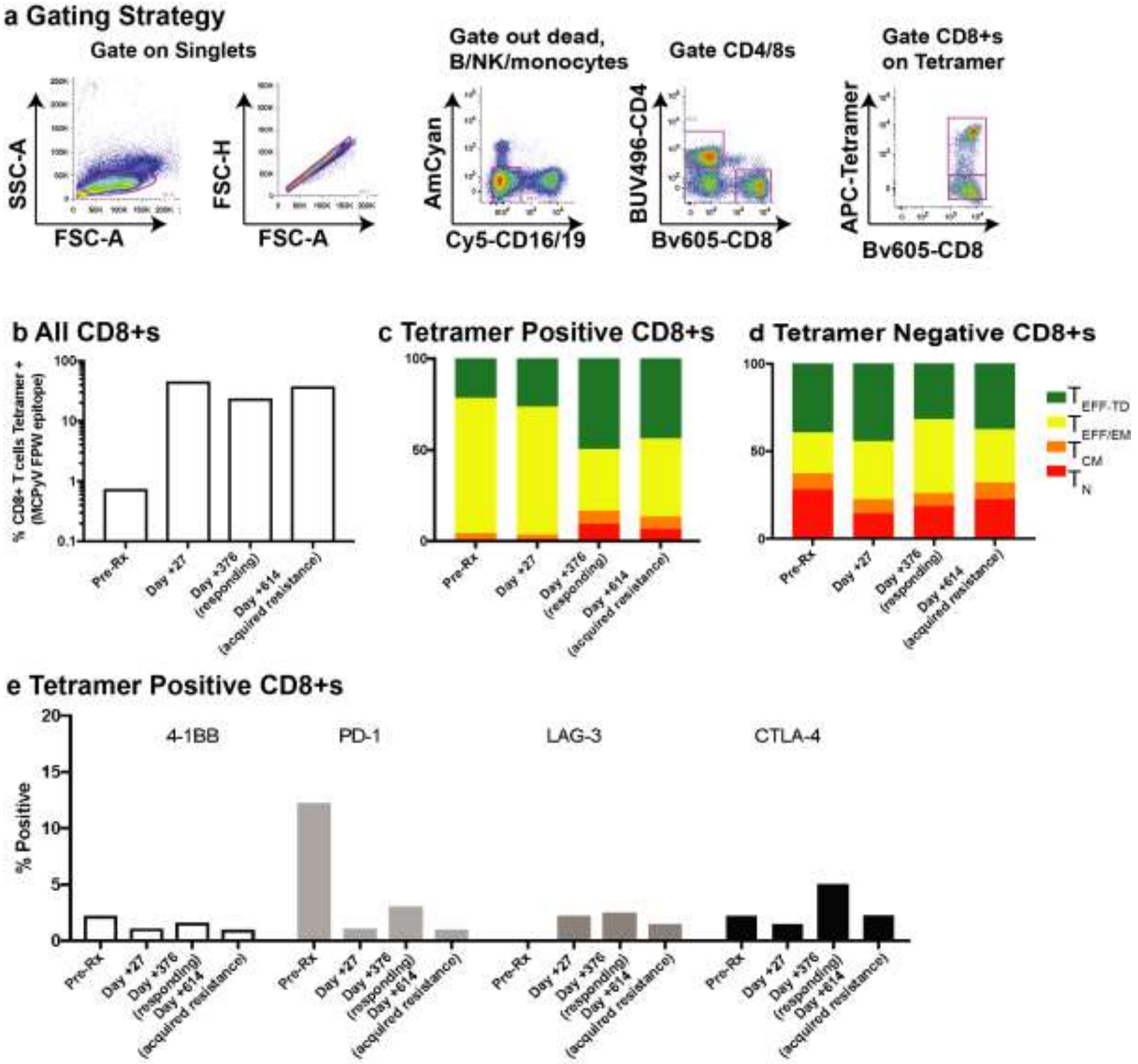

Supplementary Fig. 4:
Flow cytometry of peripheral blood at key clinical time points.
Data from the discovery patient (2586-4) are shown. For panels **b-f**, key time points are represented pre-treatment, post-T cells prior to response (d+27), during response (d+376), and at time of acquired resistance (d+614). Tetramer refers to HLA-B3502- $sT_{83\text{-}91}$ tetramer, corresponding to the infused specificity of autologous *ex vivo* expanded virus specific T cells. **a** Gating strategy: shown from left to right. **b** Percent $CD8^+$ cells tetramer positive : MCPyV-FPW/HLA-B3502. **c** Tetramer Positive $CD8^+$ T cell differentiation: $T_{Eff\text{-}TD}$ (T effector – terminally differentiated) = $CCR7^-CD45RO^-$, $T_{EFF/EM}$ (T effector – effector memory) = $CCR7^-CD45RO^+$, T_{CM} (T central memory) =$CCR7^+$CD45RO+, and T_N (naïve) =$CCR7^+$ $CD45RO^-$. A relative expansion of $tetramer^+$ effectors was observed at time of treatment response. As expected, most $tetramer^+$ CD8 cells were antigen-experienced (not naïve). **d** Tetramer negative $CD8^+$ T cell differentiation: status at the same time points. **e** Expression of activation and immune checkpoint proteins: $tetramer^+$ $CD8^+$ T cells in peripheral blood are shown.

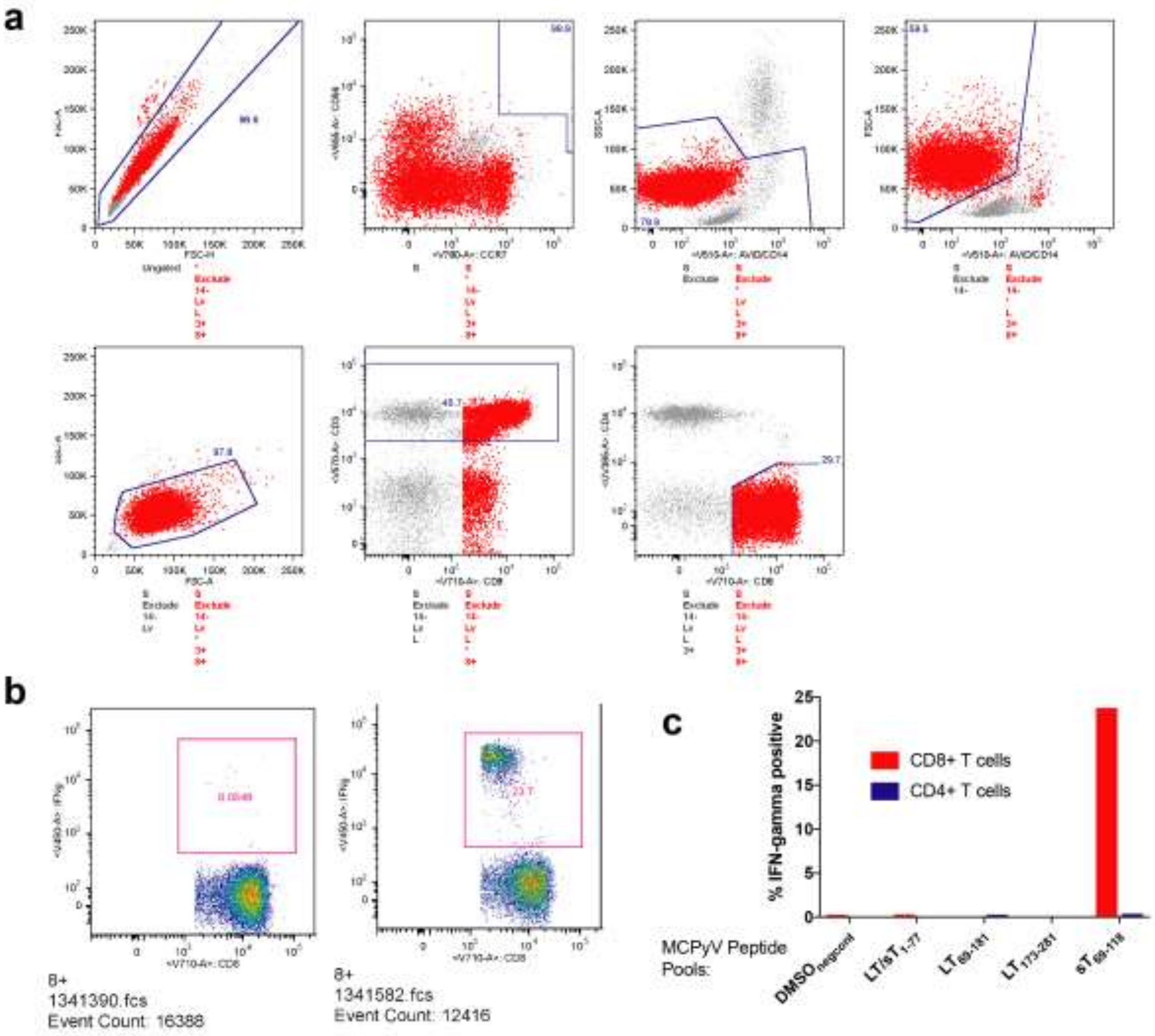

Supplementary Fig. 5:
Absence of epitope spreading to other epitopes of MCPyV at clinical response (d+334).
Data from the discovery patient (2586-4) are shown. At time of clinical response, patient PBMC were stained for intracellular interferon-gamma following culture with pools of peptides tiling across the entire expressed portion of Merkel cell polyomavirus (MCPyV) large and small T antigen to evaluate in an HLA-agnostic fashion for T cell responses to Merkel cell polyomavirus other than those associated with the T cells infused. The detected CD8$^+$ response is to the small T antigen peptide pool (reflecting infusion of CD8$^+$ cells restricted to the sT$_{83\text{-}91}$ HLA-B3502 epitope)**. a** Gating strategy: CD8$^+$ cells are shown. A similar approach was used for CD4$^+$ cells**. b** Example positive and negative sample: PBMC exposed to DMSO (left panel, negative control) and the small T antigen pool containing the known immunizing epitope (right panel, positive control) are shown. **c** CD8$^+$ and CD4$^+$ responses across peptide pools: Amino acid ranges for the large and small T antigen spanned by peptide pools are as indicated. CD8$^+$ responses are shown in red, and CD4$^+$ responses in blue.

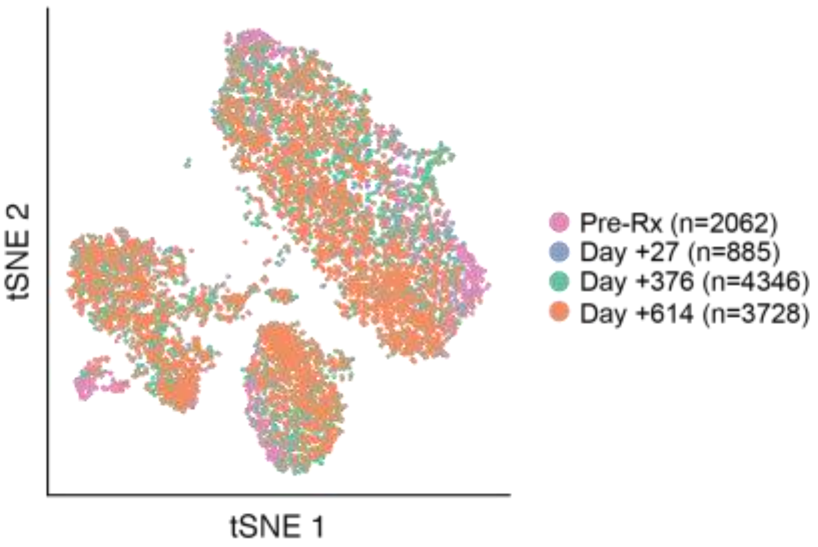

Supplementary Fig. 6:
Absence of significant batch effect between peripheral blood time points
scRNAseq data from 11,021 cells from each of four PBMC time points from the featured discovery patient (2586-4) are shown (tSNE visualization of cluster). Cell populations overlay each other at all time points, suggesting no major batch specific effects on expression analysis. Day +376 represents during response and day +614 after acquired resistance.

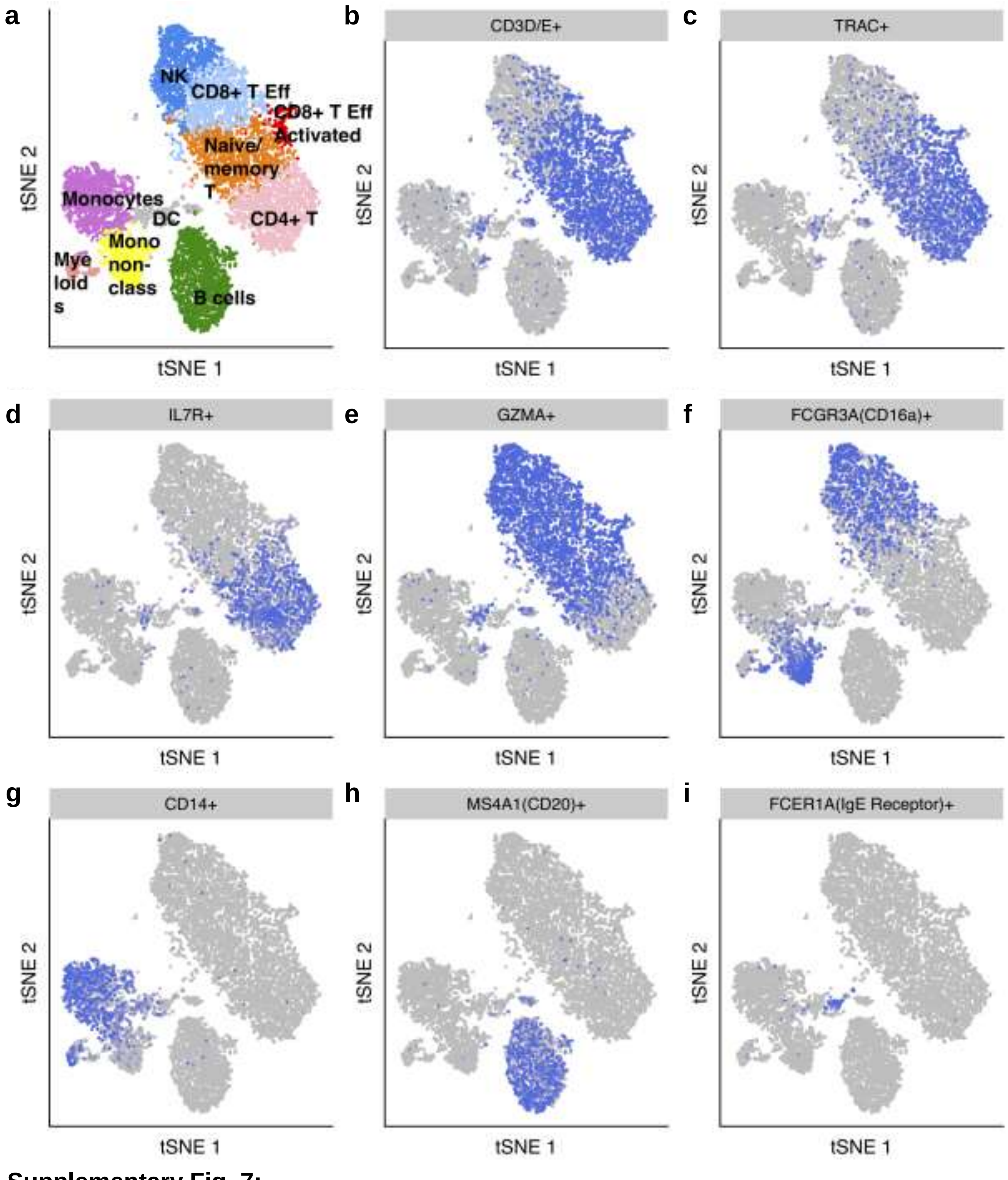

Supplementary Fig. 7:
Identification of cell populations in peripheral blood
Validation patient 2586-4 is shown. N=11021 cells, each represented with a dot, from all four time points. **a** Clusters: clusters were identified by principal component analysis and visualized with tSNE. **a** is reprinted from **Fig. 2a** for clarity. **b-i** Selection of key markers: that assisted in confirmation of cluster identification. Gene names are shown. Blue color = expressed, with darker blue indicating greater expression.

a MCC Markers

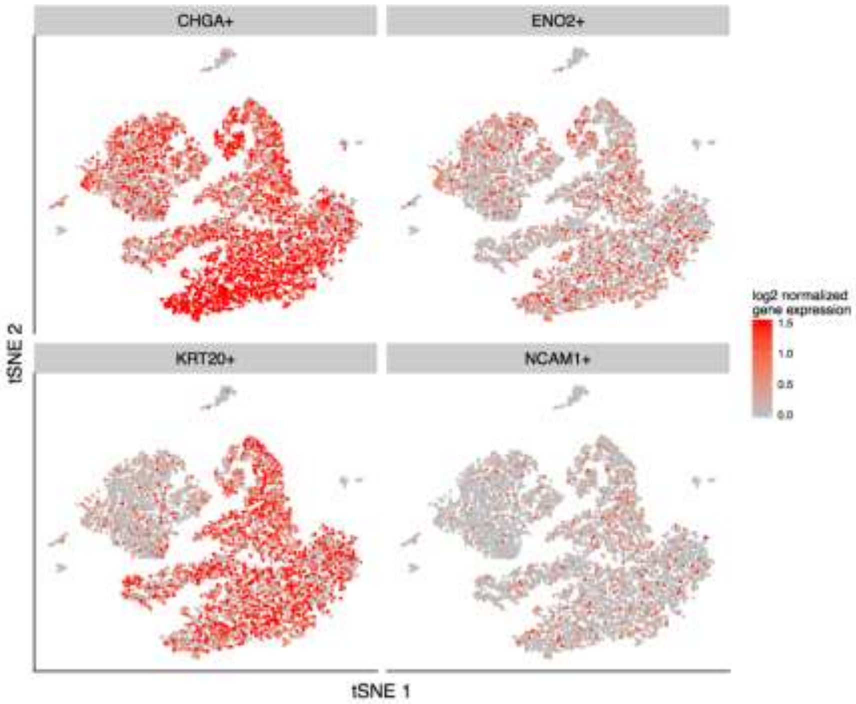

b TIL/stromal/microenvironment markers

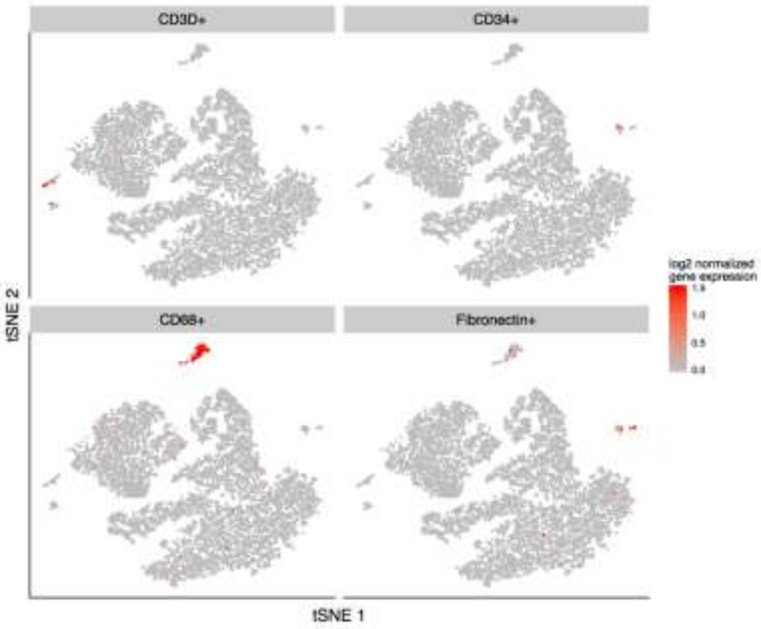

Supplementary Fig. 8:
Identification of clusters in tumor specimens
Patient 2586-4; discovery patient, selected markers are shown. **a** Canonical MCC markers: chromogranin A, neuron-specific enolase, cytokeratin 20, and CD56 to confirm tumor clusters. **b** Identification of TIL/stromal/microenvironment clusters: Gene names are shown. Red = expressed. N = 7431 cells.

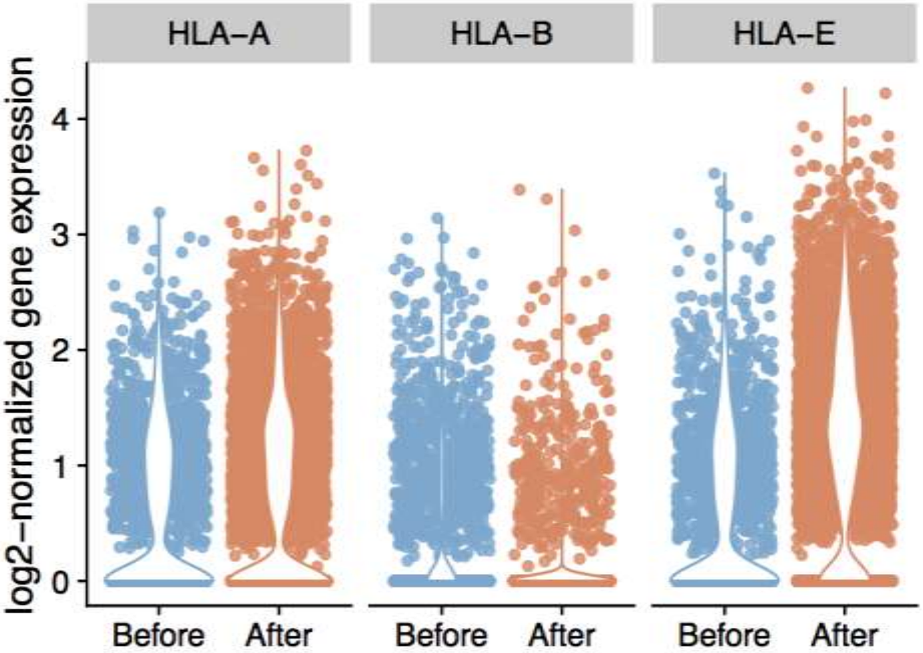

Supplementary Fig. 9:
scRNAseq violin plots for HLA-A, HLA-B, and HLA-E
Dots represent tumor cells from discovery patient (2586-4). Expression of HLA-A, -B, and E- are compared in the before treatment (n = 1984, blue) and post-acquired resistance (n = 5131, orange) tumor cells, shown as a violin plot of the data represented in **Figure 4d** (This supplemental figure contains additional information about degree of expression). Note the wide base of the HLA-B in the acquired resistance time point condition, indicates the majority of cells have no HLA-B expression. Higher = more expression. HLA-B and HLA-E were significantly differentially expressed (Methods), whereas HLA-A was not.

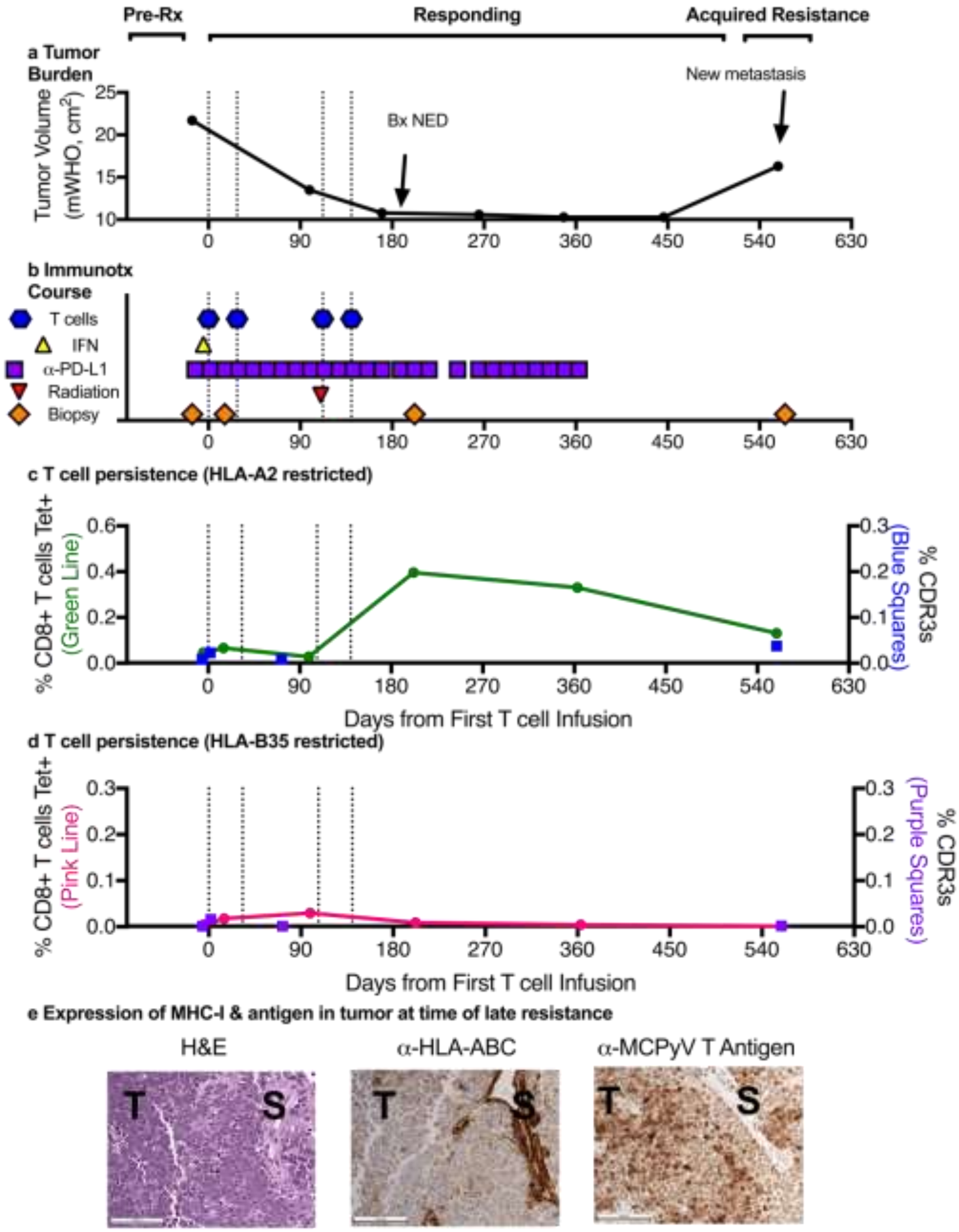

Supplementary Fig. 10:
Acquired resistance to combination immunotherapy
Validation patient (9245-3) is shown. T cell infusions indicated by dashed lines. **a** Clinical course. **b** Immunotherapy treatments. **c** T cell persistence:T cells restricted to HLA-A2/MCPyV $cT_{15\text{-}23}$. CD8^{+} T cell persistence by tetramer are shown on on left axis (green line) and frequency of dominant infused clone by CDR3-beta sequencing on right axis (blue squares). **d** T cell persistence: T cells restricted to HLA-B35/MCPyV-$sT_{83\text{-}91}$. CD8^{+} T cell persistence by tetramer is on left axis (pink line) and frequency of dominant infused clone by CDR3-beta sequencing on right axis (purple squares). No perisistence was observed beyond 180 days by either measure. **e** Evaluation of tumor at acquired resistance: IHC demonstrating expression of MHC class I (pan-HLA–ABC) in tumor (T) and stroma (S) and MCPyV T antigen in tumor at acquired resistance (day +565). Scale bar = 100 micrometers.

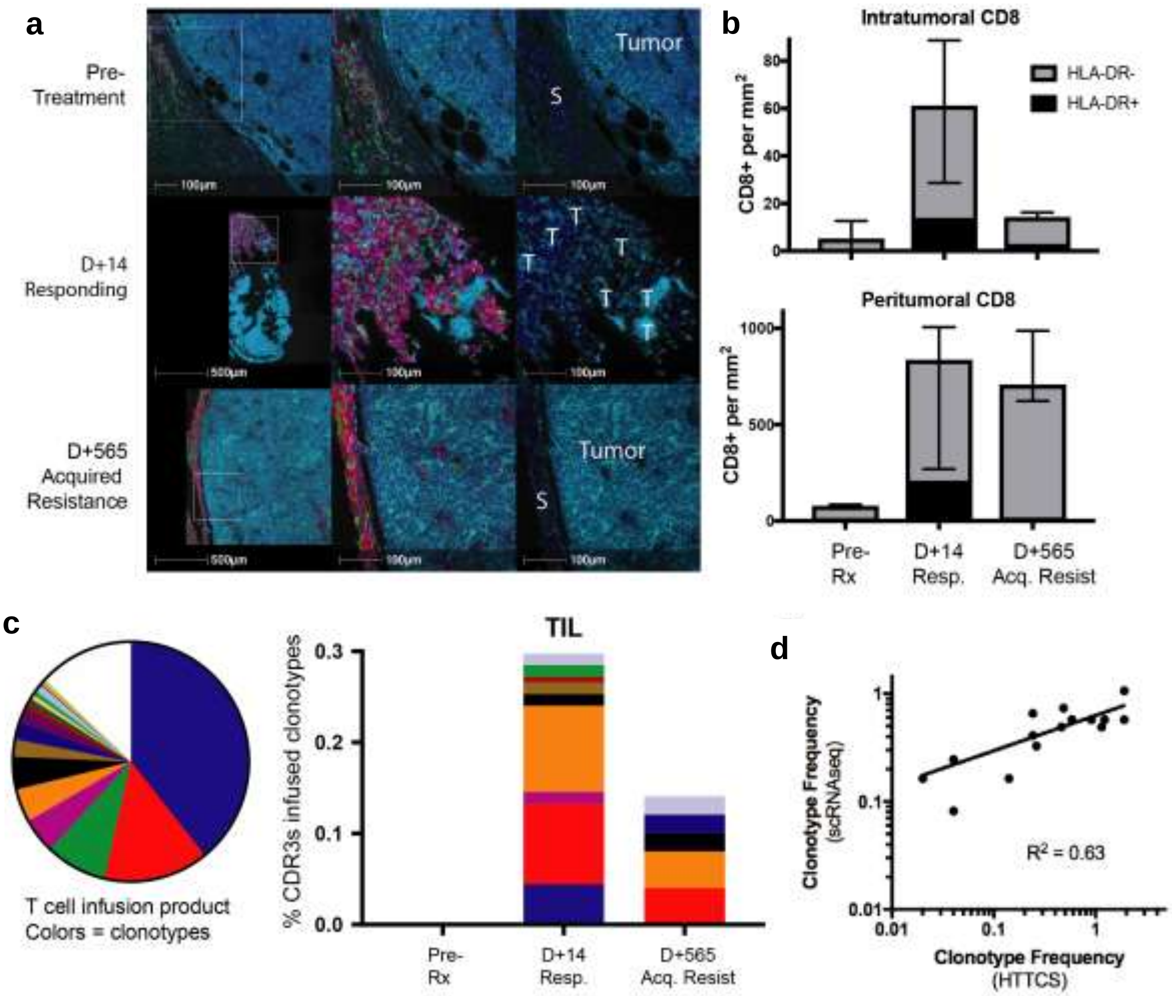

Supplementary Fig. 11:
Activated CD8+ T cell infiltration into tumor at the time of treatment response
Validation patient (9245-3) is shown **a** Multiplex immunohistochemistry: showing representative peritumoral and intratumoral $CD8^+$ infiltrates at key time points including response (day +14) and acquired resistance (day +565). Left panel and middle panel show two magnifications, with magnified area shown by boxes. Right panel shows expression of CD56 only, to better allow delineation of fragments of tumor at time point of response, which are now surrounded by advancing T cells. Dark blue: DAPI (nuclei), Light blue = CD56 (MCC tumor), Red = $CD3^+CD8^-$, Green = $CD8^+$, Purple = HLA-DR. Scale bars and lengths are indicated on the bottom of each image, and are 100 microns for all center and right panels. **b** Quantification of density of intratumoral and peritumoral CD8+ cells: Error bars represent range of each of three scored scan fields, a representative example of which is shown in panel **a**. **c** Detection of infused HLA-A2 restricted T cell clonotypes: in tumor at time of treatment response by sequencing of TCR CDR3-beta. Time points as indicated. Each of the 20 infused MCPyV specific clonotypes is shown by separate color, as indicated at left. **d** Correlation between clonotype frequency (percent CDR3s) between high-throughput T cell sequencing and scRNAseq. Fifteen clonotypes are shown (top 10 most highly expressed, and then representative examples at frequencies of 0.3, 0.2, 0.15, 0.1 by scRNAseq as well as top detected MCPyV specific clonotype CDR3). Separate fragments of the relapsed tumor nodule were used for DNA isolation and single cell digest creation, respectively.

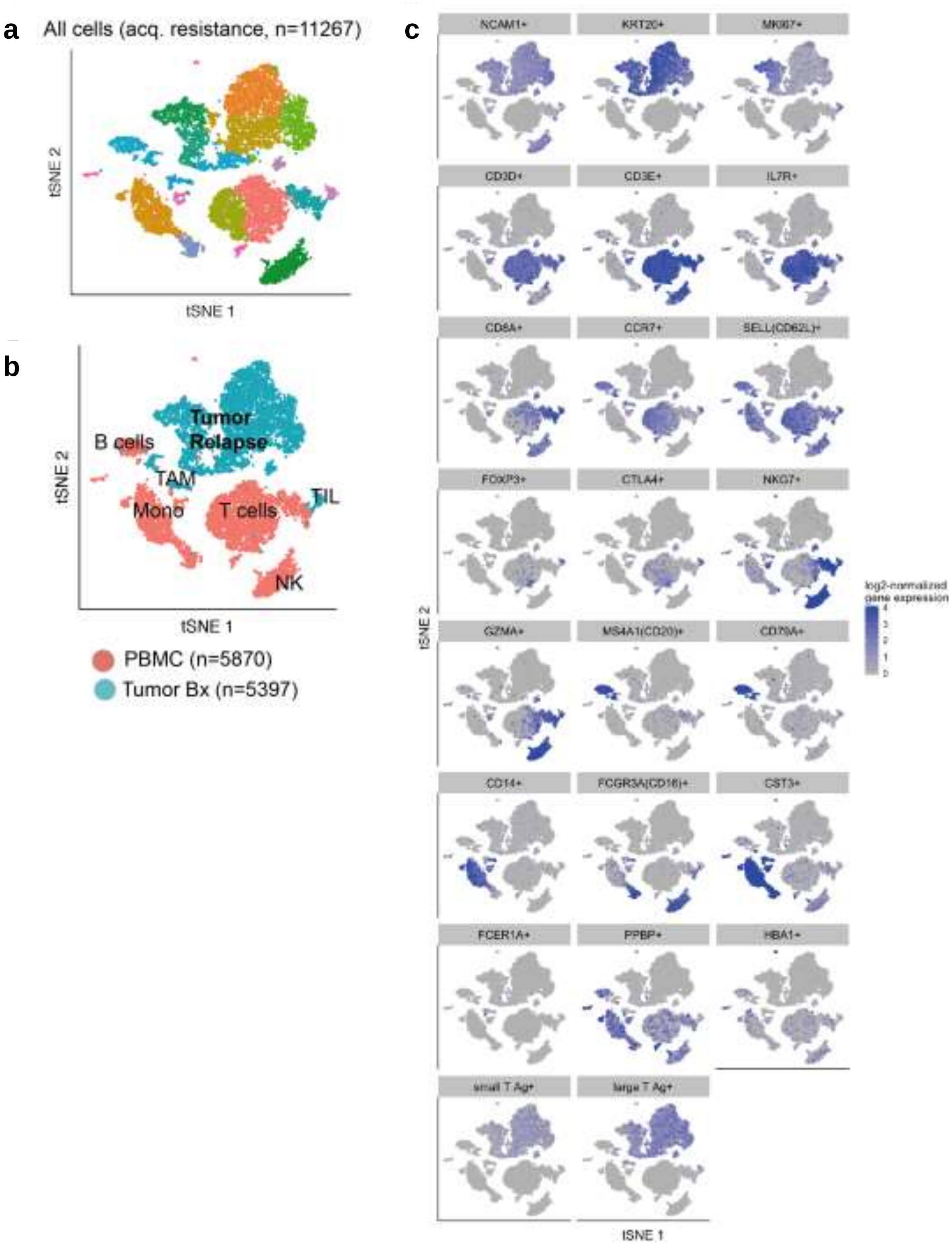

Supplementary Fig. 12:
Single cell RNA sequencing of validation patient (9245-3).
For this patient, only tumor from late relapse/acquired resistance was available. Tumor (day +565) and peripheral blood (day +559) from the same late resistance time point were run. **a** Clustering and **b** Sample ID. **b** is reproduced from Fig. 4f for clarity. **c** Key genes used to help identify clusters. NCAM1(CD56), KRT20 (CK20) and MCPyV Large and Small T antigen help to define tumor clusters. Other TIL and peripheral blood populations were defined using the markers as listed. For each gene, the gene name is listed in grey bar and degree of expression indicated by blue color (darker blue = more expression).

Supplementary Tables:

Event	CTCAE Grade	Relationship to Treatment	Expected?	Duration of AE
Anemia	3	Possible	Unexpected	27 days
Fevers and chills	3	Related	Expected	1 day (first infusion); 1 day (second infusion)
Lymphopenia	4	Related	Expected	7 days (first infusion); 4 days (second infusion)

Supplementary Table 1:
Toxicities of T cell therapy – Patient 2586-4
Grade 3-4 treatment toxicities that were possibly or definitively related to T cell therapy are listed, including event, degree and relationship to treatment. All toxicities were transient and fully resolved. The first febrile episode required hospital admission for <48 hours to the general ward and was managed with supportive interventions (fluids, acetaminophen); the second event (second infusion) did not require hospital admission and was managed on an outpatient basis. Anemia was unexpected and possibly linked to radiation.

Coordinates (HG19)	Ref (HG19)	Alt (observed in patient)	Location In Gene	Germline (PBMC)	Tumor Pre-Treatment	Tumor Acquired Resistance
31324996	G	C	Upstream/Promoter	C/C Hom	C/C Hom	C/C Hom
31324953	C	T	5' UTR	T/T Hom	T/T Hom	T/T Hom
31324931	A	C	Exonic	C/C Hom	C/C Hom	C/C Hom
31324925	A	G	Exonic	G/G Hom	G/G Hom	G/G Hom
31324911	C	G	Exonic	C/G Het	C/G Het	C/G Het
31324895	G	C	Exonic	C/C Hom	C/C Hom	C/C Hom
31324892	G	C	Exonic	C/C Hom	C/C Hom	C/C Hom
31324888	G	T	Exonic	T/T Hom	T/T Hom	T/T Hom
31324887	G	C	Exonic	C/C Hom	C/C Hom	C/C Hom
31323945	A	C	Exonic	C/C Hom	C/C Hom	C/C Hom
31323838	C	G	Intronic	G/G Hom	G/G Hom	G/G Hom
31322996	C	T	Exonic	T/T Hom	T/T Hom	T/T Hom
31322980	C	T	Exonic	C/T Het	C/T Het	C/T Het
31322911	C	T	Exonic	C/T Het	C/T Het	C/T Het
31322888	A	G	Exonic	G/G Hom	G/G Hom	G/G Hom
31322303	C	G	Exonic	C/G Het	C/G Het	C/G Het

Supplementary Table 2:
Results of whole exome sequencing for HLA-B/NM005514 (Patient 2586-4)
No tumor-associated insertions, deletions, SNPs, or loss of heterozygosity (which would indicate allelic loss) was detected in HLA-B or the sequenced portions of the HLA-B promoter region by whole exome sequencing. Please see Table S2 for comprehensive results of whole exome sequencing and all observed exonic somatic coding mutations. Patient 2586-4 (discovery) patient is shown.

Gene Name	P	Fold Change	CI - Hi	CI - Low
Genes more highly expressed in red cluster than blue cluster				
ACTB	1.27E-07	-0.43	-0.28	-0.58
ACTG1	1.81E-28	-0.69	-0.41	-0.96
ARHGDIB	5.14E-07	-0.38	-0.19	-0.58
CD52	1.00E-68	-1.59	-1.39	-1.80
CORO1A	1.17E-10	-0.42	-0.21	-0.63
COTL1	3.84E-13	-0.41	-0.26	-0.55
GAPDH	6.34E-22	-0.64	-0.43	-0.84
GZMA	5.46E-11	-0.48	-0.27	-0.69
IL32	6.75E-21	-0.80	-0.58	-1.02
LGALS1	2.73E-07	-0.56	-0.33	-0.80
MT-CO1	9.73E-08	-0.40	-0.26	-0.53
MT-CO3	2.76E-26	-0.76	-0.63	-0.90
MT-CYB	7.11E-06	-0.38	-0.23	-0.53
MT-ND1	1.81E-06	-0.40	-0.27	-0.54
MYL6	2.12E-05	-0.39	-0.22	-0.56
S100A10	7.65E-12	-0.59	-0.38	-0.80
S100A4	4.03E-27	-0.82	-0.64	-1.01
S100A6	7.11E-12	-0.58	-0.39	-0.76
TMSB10	1.30E-08	-0.38	-0.26	-0.51
TRAC	2.07E-24	-0.71	-0.54	-0.88
Genes more highly expressed in light blue cluster than red cluster				
B2M	7.94E-13	0.41	0.51	0.30
CMC1	1.25E-21	0.76	0.88	0.64
CST7	3.68E-11	0.63	0.78	0.47
CTSW	4.36E-12	0.59	0.71	0.47
FCGR3A	4.19E-27	0.41	0.46	0.35
FGFBP2	2.11E-15	0.73	0.86	0.60
GNLY	2.18E-06	0.67	0.98	0.35

GSTP1	1.16E-10	0.39	0.48	0.30
GZMB	9.42E-09	0.63	0.80	0.46
GZMH	2.04E-14	0.81	0.98	0.65
H3F3A	2.02E-05	0.39	0.63	0.16
HLA-B	1.25E-11	0.46	0.59	0.34
HLA-C	1.16E-07	0.43	0.59	0.26
HLA-DPB1	8.07E-16	0.40	0.48	0.32
ID2	3.46E-13	0.40	0.49	0.32
IFITM2	3.18E-10	0.51	0.67	0.36
ITGB2	6.28E-12	0.41	0.50	0.32
MALAT1	5.35E-98	1.62	1.77	1.47
NKG7	1.60E-79	1.68	1.86	1.50
RPL27	2.83E-05	0.41	0.64	0.18
SRGN	1.62E-08	0.41	0.53	0.29
TRDC	1.06E-27	0.60	0.67	0.53
TXNIP	4.91E-10	0.50	0.64	0.35
TYROBP	2.54E-28	0.79	0.88	0.70
UBB	4.90E-05	0.39	0.56	0.21

Supplementary Table 3:
List of significantly differentially expressed genes between PBMC derived CD8+ T cells in the blue effector/EM cluster (n = 429) to those in the red activated cluster (n = 170) at the time point of treatment response (day +376).
Patient 2586-4 (discovery) patient is shown. Genes organized by whether higher expression in red cluster (top) or blue cluster (bottom) and then alphabetically within categories. Negative fold change = more highly expressed in red cluster. N=45 DEGs; see methods for determination of DEGs.

Gene	Chromosome	Position	Reference	Pre-Tr	Relapse
HLA-A	6	29910358	C	G	G
HLA-A	6	29910558	T	C	C
HLA-A	6	29910572	C	T	T
HLA-A	6	29910716	C	G	G
HLA-A	6	29910717	A	G	G
HLA-A	6	29910730	T	A	A
HLA-A	6	29910742	G	C	C
HLA-A	6	29910752	G	C	C
HLA-A	6	29911056	A	G	G
HLA-A	6	29911063	T	G	G
HLA-A	6	29911064	A	G	G
HLA-A	6	29911092	G	T	T
HLA-A	6	29911114	G	A	A
HLA-A	6	29911119	G	T	T
HLA-A	6	29911154	C	A	A
HLA-A	6	29911198	T	C	C
HLA-A	6	29911207	G	A	A
HLA-A	6	29911228	A	T	T
HLA-A	6	29911256	T	G	G
HLA-A	6	29911901	C	G	G
HLA-A	6	29911928	C	G	G
HLA-A	6	29911931	A	G	G
HLA-A	6	29911970	G	A	A
HLA-A	6	29912108	G	C	C
HLA-A	6	29912280	T	C	C
HLA-A	6	29912333	C	T	T
HLA-A	6	29912856	A	T	T

Supplementary Table 4:
Results of whole exome sequencing for HLA-A (validation Patient).
HLA-A sequence was identical in pre-treatment and post-relapse tumor samples for the validation patient (9245-3). Coding SNPs are shown. Reference human genome reference 19.
