# Supplementary material for: Acquired cancer resistance to combination immunotherapy from transcriptional loss of class I HLA
Source: Nat Commun. 2018 Sep 24;9:3868. doi: 10.1038/s41467-018-06300-3 (PMC6155241; doi:10.1038/s41467-018-06300-3)
Supplement: Supplementary file 3 — Description of Additional Supplementary Files [file 41467_2018_6300_MOESM3_ESM.pdf]

Description of Additional Supplementary Files

File Name: Supplementary Data 1
Description: **Somatic Mutations Identified by Whole Exome Sequencing.** Data is from discovery patient (2586-4). All identified somatic mutations (present in tumor but not peripheral blood) that are exonic and resulted in coding change are listed. Columns identify whether the mutation was detected in the pre-treatment tumor specimen, acquired resistance specimen, or both.

File Name: Supplementary Data 2
Description: **List of differentially expressed genes (DEGs) between tumor cells at the pre-treatment and acquired resistance tumors.** Data is from discovery patient (2586-4) single cell RNA sequencing data. Genes are ordered from genes most highly over-expressed in the acquired resistance tumor to genes most down-regulated in the acquired resistance tumor. Upregulated and downregulated genes are included on different spread-sheet tabs. Negative fold change = more highly expressed in the acquired resistance tumor, positive fold change = more highly expressed in the pre-treatment tumor. All 255 statistically significant DEGs (Methods) are listed.

File Name: Supplementary Data 3
Description: **R script to generate tSNE plots for Tumor and PBMC for discovery patient.**

File Name: Supplementary Data 4
Description: **R script to generate tSNE plots for Tumor and PBMC for validation patient.**
